# Periostin Exon-21 Antibody Neutralization of Triple-Negative Breast Cancer Cell-Derived Periostin Regulates Tumor-Associated Macrophage Polarization and Angiogenesis

**DOI:** 10.3390/cancers13205072

**Published:** 2021-10-11

**Authors:** Tatsuya Fujikawa, Fumihiro Sanada, Yoshiaki Taniyama, Kana Shibata, Naruto Katsuragi, Nobutaka Koibuchi, Kaori Akazawa, Yuko Kanemoto, Hidehito Kuroyanagi, Kenzo Shimazu, Hiromi Rakugi, Ryuichi Morishita

**Affiliations:** 1 Department of Clinical Gene Therapy, Osaka University Graduate School of Medicine, Osaka 565-0871, Japan; fujikawa@cgt.med.osaka-u.ac.jp (T.F.); sanada@cgt.med.osaka-u.ac.jp (F.S.); taniyama@cgt.med.osaka-u.ac.jp (Y.T.); shibata@cgt.med.osaka-u.ac.jp (K.S.); katsuragi@cgt.med.osaka-u.ac.jp (N.K.); koibuchi@cgt.med.osaka-u.ac.jp (N.K.); 2 Department of Geriatric Medicine and Nephrology, Osaka University Graduate School of Medicine, Osaka 565-0871, Japan; rakugi@geriat.med.osaka-u.ac.jp; 3 Department of Breast and Endocrine Surgery, Osaka University Graduate School of Medicine, Osaka 565-0871, Japan; k.ooi0816@onsurg.med.osaka-u.ac.jp (K.A.); y.kanemoto@onsurg.med.osaka-u.ac.jp (Y.K.); kshimazu@onsurg.med.osaka-u.ac.jp (K.S.); 4 Department of Biochemistry, Graduate School of Medicine, University of the Ryukyu, Okinawa 903-0213, Japan; hidehito@med.u-ryukyu.ac.jp

**Keywords:** periostin, triple-negative breast cancer, tumor-associated macrophage

## Abstract

**Simple Summary:**

Despite remarkable advances in breast cancer treatment, few strategies other than standard cytotoxic chemotherapy are available for patients with triple-negative breast cancer (TNBC) due to the lack of therapeutic target molecules. TNBC is still the most aggressive subtype, with a high risk of recurrence and metastasis within 2 years after initial treatment. Thus, there is an unmet medical need to develop new treatments for metastatic and recurrent TNBC patients. In this study we tested a new antibody, targeting extracellular periostin protein alternative splicing variants, which are induced by conventional chemotherapy or during the process of endothelial mesenchymal transition. This antibody reduced periostin-secreting TNBC in a mouse xenograft model, accompanied by a decrease in the number of M2 tumor-associated macrophages and tumor vessels. Periostin alternative splicing variants might be a specific and safe therapeutic target in patients with TNBC.

**Abstract:**

Periostin (Pn) is involved in multiple processes of cancer progression. Previously, we reported that Pn expression is correlated with mesenchymal tumor markers and poor prognosis in triple-negative breast cancer (TNBC). In the TNBC xenograft model, chemotherapy increased expression of a Pn alternative splicing variant (ASV) with exon 21, and administration of the neutralizing antibody against Pn with exon 21 (Pn-21 Ab) overcame chemoresistance with a reduction in the mesenchymal cancer cell fraction. In the present study, the role of Pn ASV with exon 21 in TNBC progression has been addressed. We first established a stable cell line carrying a fluorescence-based splicing reporter. Pn-positive TNBC has higher expression of genes related to tumor-associated macrophage (TAM) recruitment and ECM-receptor interaction than Pn-negative cells. In a xenograft model, only Pn-positive cells initiated tumor formation, and the Pn-21 Ab suppressed tumor cell growth, accompanied by decreased M2 TAM polarization and the number of tumor vessels. These data suggest that cancer cell-derived Pn ASV educates TAMs and regulates angiogenesis, which in turn establishes a microenvironmental niche that is supportive of TNBC.

## 1. Introduction

Despite remarkable advances in cancer treatment approaches, breast cancer is still the main cause of cancer-related death among females worldwide, with an estimated 2.1 million new cases and 626,679 deaths in 2018 [1]. Statistically, breast cancer accounts for almost 1 in 4 newly diagnosed cancer cases and 15% of cancer deaths among women. Its principal cause is the acquisition of chemoresistance, mainly in advanced disease stages [2]. The discovery of hormone receptors and a mutated gene named HER2 led to the innovation of new targeted therapies for the luminal and HER2 subtypes [3]. Nonetheless, due to the lack of therapeutic target molecules, few strategies other than standard cytotoxic chemotherapy are available for patients with triple-negative breast cancer (TNBC). TNBC is still the most aggressive subtype, with a high risk of recurrence and metastasis within 2 years after initial treatment [4]. Thus, there is an unmet medical need to develop new treatments for metastatic and recurrent TNBC patients.

We previously showed that extracellular matrix periostin (Pn) protein expression is associated with the epithelial-to-mesenchymal transition (EMT) gene signature, especially in breast cancer cells [5]. Among the breast cancer subtypes, the level of Pn expression is well correlated with poor prognosis among patients with TNBC. In the TNBC xenograft model, Pn is induced by conventional chemotherapy in tumor cells and expands the mesenchymal tumor-cell population, leading to chemoresistance. Furthermore, a chemotherapy-induced cancer-specific ASV of Pn, which contains Pn exon 21, is secreted into the extracellular space, and the application of the neutralizing antibody targeting Pn exon 21 overcomes TNBC recurrence following conventional chemotherapy. These data suggest that Pn with exon 21 has a distinct role in supporting cancer development, although the data regarding the function of Pn ASV including exon 21 is limited [5,6].

In this study, using a Pn splicing reporter, we found that SUM159PT TNBC cells contain two populations: (1) a population expressing higher levels of Pn (Pn-positive cells), including an ASV with exon 21, and genes related to extracellular matrix proteins, focal adhesion proteins, and inflammatory cytokines recruiting tumor-associated macrophages (TAMs), and (2) a population secreting much less Pn (Pn-negative cells). In a xenograft model of TNBC, only the Pn-positive population can initiate tumors. Application of a neutralizing antibody specifically targeting Pn exon 21 halts disease progression without toxicity. This favorable effect is accompanied by reduced M2 TAM accumulation and tumor vessels. In vitro experiments confirm that recombinant Pn with exon 21 reduces M1 TAM markers but enhances M2 TAM markers in mouse THP-1 and U937 cells, as well as human CD14+ peripheral blood mononuclear cells.

Taken together, these data show that TNBC secreting a specific Pn ASV induces M2 TAM polarization and tumor vessel formation, establishing a microenvironmental niche supportive of breast cancer.

## 2. Materials and Methods

### 2.1. Anti-Human Periostin Antibody

In order to raise the mouse monoclonal antibody against exon 21 of human Pn, the exon-21 peptide was synthesized at Oriental Yeast Co., Ltd. The antibody was generated in immunized mice. Mouse monoclonal Pn antibody against exon 12 was obtained from AdipoGen life science (San Diego, CA, USA, cat # AG-20B-0033-C100). We tested the specificity of this antibody by dot plot using peptides of each exon.

### 2.2. Cell Culture

SUM 159, BT549 and MCF10 DCIS human triple-negative breast cancer cell lines were obtained from Asterand (Detroit, MI, USA), American Type Culture Collection (Manassas, VA, USA) and Wayne State University, respectively. SUM159PT cells were cultured in Ham’s F-12 medium supplemented with 5% fetal bovine serum (FBS), 10 mM HEPES, 5 μg/mL insulin, and 1 μg/mL hydrocortisone. BT549 cells were cultured in Dulbecco’s Modified Eagle Medium (DMEM) supplemented with 10% FBS. MCF10DCIS cells were cultured in DMEM/F12 media with 5% horse serum, 20 ng/mL EGF, 10 μg/mL insulin, 0.5 μg/mL hydrocortisone, and 100 ng/mL cholera toxin. Cell migration was measured by using Oris™ Cell Migration Assays (Funakoshi, Tokyo, Japan, cat # CMA1–101) according to the manufacturer’s instructions, human CD14+ peripheral blood mononuclear cells (PBMCs) from LONZA (Basel, Switzerland) were grown in X-VIVO™ 15 serum free medium. PBMCs were differentiated using 10 ng/mL granulocyte-macrophage colony stimulating factor (GM-CSF, R & D systems, Minneapolis, MN, USA, cat # 215-GM-010) for 6 days, then media was replaced with fresh X-VIVO™ 15 serum-free medium for 24 hours. U937 and THP-1 cells from American Type Culture Collection (Manassas, VA, USA) were grown in RPMI 1640 + 10% FBS. 2.0 × 10^6^ U937 or THP-1 cells/well in 6-well plates were stimulated with 100nM PMA for 48 hours, then media was replaced with fresh serum-free media for 24 hours. 500 ng/mL full-length recombinant periostin (Pn-1; R & D systems, MN, USA, cat # 3548-F2–050) or periostin lacking exon 17 and 21 (Pn-4; SinoBiological, Chesterbrook, PA, USA, cat # 10299-H08H) were administered to the media, and cells (PBMCs, U937 and THP-1) were harvested after 24 hours for the RNA analysis.

### 2.3. Human Periostin Exon-21 Splicing Reporter

To examine periostin exon-21 inclusion and exclusion patterns, florescent reporter system was constructed in the pFRT/lacZeo vector (ThermoFisher Science Inc, Boston, MA, USA, cat # V601520) as shown in Figure S5. To construct the periostin exon-21 reporter cassettes, the periostin genomic DNA fragment spanning from intron 20 to exon 21 to intron 21 was cloned in between EGFP cDNA, followed by mCherry cDNA with different frames under the control of human cytomegalovirus (CMV) promoter. To adjust the reading frame of mCherry, we introduced a single nucleotide into exon 21 in the reporter. The reporter minigenes for periostin exon 21 were introduced into SUM159PT or BT549 TNBC cells using Flp-In™ System (ThermoFisher science Inc, MA, USA, cat # K601002) for the generation of stable cell lines.

### 2.4. RNA Sequence

RNA sequence analysis was performed using double-positive and GFP-positive SUM159PT cells. For differential expression analysis, the data on gene level counts were generated using featureCounts version 0.5.4p3 [7]. Differentially expressed genes (DEGs) were identified using the read count data by the R software package TCC [8]. Calculating normalization factors used the DEGES/edgeR method. The q-value was analyzed from the *p* value using the *p*.adjust function in the R package and the default settings. DEGs were identified based on a q-value threshold of less than 0.05.

### 2.5. Cell Line-Derived Xenograft Model Studies in Mice

All experimental procedures were reviewed and approved by the Institutional Animal Committee at the Department of Veterinary Science of Osaka University School of Medicine (approved number; 30-023-015) and followed the recommendations of the guidelines for animal experimentation at research institutes (Ministry of Education, Culture, Sports, Science and Technology, Chiyoda, Japan), guidelines for animal experimentation at institutes (Ministry of Health, Labor and Welfare, Tokyo, Japan), and guidelines for proper conduct for animal experimentation (Science Council of Japan). In all studies, female athymic nude mice at the age of 8 weeks were used. Mice were inoculated sc with 5 × 10^6^ SUM159PT cells suspended in 0.1 mL Matrigel-PBS. Tumor volumes were monitored every 3–4 days by caliper measurement of the length, width, and height and were calculated by using the following formula: tumor volume (mm^3^) = 1/2 × length (mm) × width (mm)^2^. One groups of animals received Pn-21 Ab (10 mg/kg) or isotype control IgG (10 mg/kg, BioXCells, Lebanon, NH, USA) 2 times per week, and the other group of animals were administered saline as for control group.

### 2.6. Histological Analysis

Isolated perfused tumors were fixed in buffered 10% formalin and embedded in paraffin to be sliced into horizontal 5 μm sections for immunohistochemical and immunofluorescent staining. To calculate TAM marker-positive structure and CD31-positive area in the tumor, we quantitated at least 4 view fields per tumor by using the Image J program, downloaded from the National Institutes of Health (NIH, Bethesda, MD, USA) website. The antibodies against Iba 1 (Abcam, Cambridge, UK, cat #ab5076), CD11b (BIO-RAD, Hercules, CA, USA, cat #MCA711GT), CD11c (Abcam, Cambridge, UK, cat #ab52632), Arginase 1 (Cell signaling, Danvers, MA, USA, cat # #93668), CD206 (Abcam, Cambridge, UK, cat #ab64693) and CD163 (Abcam, Cambridge, UK, cat #ab182422) were used, and ki67 was purchased from Abcam (Cambridge, UK, cat #ab16667). Anti-Nuclei Antibody, clone 235–1, Cy3 conjugate was purchased from Sigma-Aldrich (St. Louis, MO, USA).

### 2.7. Quantitative Realtime PCR

For reverse transcription, total RNA from cells or tissues was prepared as described previously [9]. RNA was quantified and integrity was confirmed. We used a High-Capacity cDNA Reverse Transcription Kit with RNase Inhibitor (ThermoFisher Scientific, Waltham, MA, USA) for synthesizing cDNA and Applied Biosystems Viia 7 (ThermoFisher Scientific, MA, USA) for detection in accordance with the manufacturer’s instructions. In each experiment, human GAPDH was amplified as a reference standard. Primer details are shown as follows.

### 2.8. Western Blot Analysis

Cell lysates or conditioned media were prepared with RIPA buffer, electrophoresed and blotted onto PVDF membranes. The membranes were incubated with primary antibodies against GAPDH (FUJIFILM, Japan, cat # 015–25473), periostin (Adipogen AG, San Diego, CA, USA, cat #AG-20B-0033-C100), Pn-21 Ab and secondary antibodies for ECL analysis.

### 2.9. PCR Primer List

Human primer list (Table 1).

### 2.10. Statistics

For statistical analysis, the values are shown as the means ± SD. All statistical analyses were performed with EZR [10], which is for R. More precisely, it is a modified version of R commander designed to add statistical functions frequently used in biostatistics. For statistical analysis of expression change of Ki67, TAM markers and CD31, the Mann–Whitney U test was used to define pairwise statistical differences. For statistical analysis of qPCR results, two-tailed Student’s *t*-test was performed. A one-way ANOVA test and Tukey–Kramer’s post hoc test were used for multiple comparisons. A *p*-value less than 0.05 was considered as statistically significant.

## 3. Results

### 3.1. Development of SUM159PT TNBC Cells with A Fluorescence-Based Periostin Exon-21 Splicing Reporter

**Figure 1:**
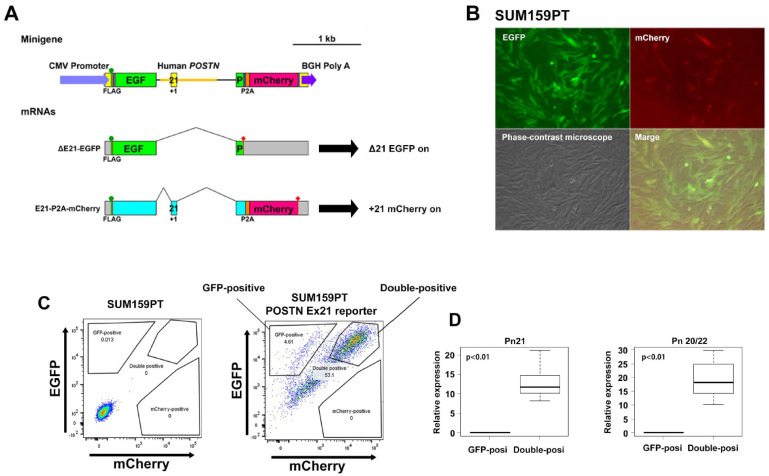

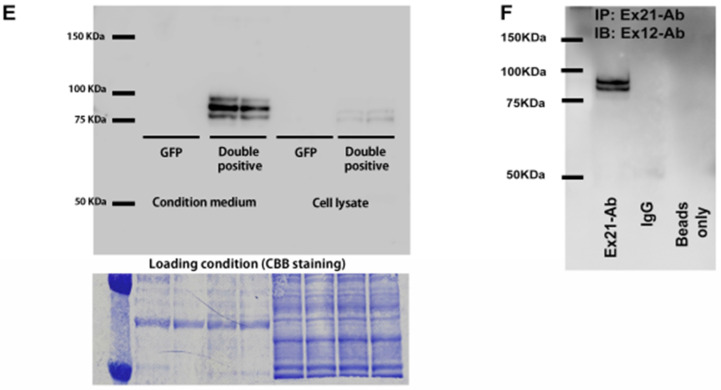
Identification of Pn-secreting SUM159PT cells. (**A**) Construction of fluorescence-based alternative splicing reporter minigenes for Pn exon 21. A genomic fragment from intron 20 to intron 21 of the POSTN gene is inserted into an artificial intron in EGFP cDNA. mCherry cDNA is connected downstream of the EGFP in another reading frame. EGFP and mCherry are expressed when POSTN exon 21 is skipped and included, respectively. (**B**) Images of SUM159PT cells with the Pn-splicing reporter. (**C**) Flow cytometry analysis of GFP and mCherry expression on SUM159PT tumor cells. SUM159PT cells with (right) or without (left) the Pn-splicing reporter. Double positivity indicates the fraction positive for both GFP and mCherry. (**D**) Pn mRNA expression in the cells from the GFP- or double-positive fraction. Pn21 and Pn20/22 indicate endogenous Pn mRNAs with exon 21 and without exon 21, respectively. *n* = 10. (**E**) Pn protein expression in conditioned media or cell lysates from GFP- or double-positive cells. Pn was detected with an antibody recognizing exon 12. Three bands indicate ASVs of Pn. Loading condition was confirmed with CBB staining. (**F**) Immunoprecipitation (IP) studies for Pn exon 21 in conditioned media from double-positive SUM159PT cells. Pn was detected with the antibody recognizing exon 12.

We previously demonstrated that Pn undergoes alternative splicing in its C-terminal region, which is devoid of known protein domains. Pn has four major alternative splicing variants (ASVs) in humans and mice (Figure S1), and ASVs that contain exon 21 are upregulated in breast cancer cells by conventional chemotherapy, including paclitaxel, doxorubicin and cyclophosphamide; however, an ASV lacking exons 17 and 21, the shortest isoform, is physiologically expressed [5]. Pn blockade with a neutralizing antibody against Pn exon 21 (Pn-21 Ab) overcomes chemoresistance by restricting mesenchymal tumor subpopulations in the MCF10DCIS xenograft model. To further investigate the role of the Pn ASVs with exon 21 in tumor progression, TNBC cell lines with high (BT549) and moderate (SUM159PT) Pn expression were used based on previous reports [11] and data available in the GOBO database (Figure S2) [12]. These TNBC cell lines are categorized as basal-B, in which cells express genes related to mesenchymal cancer cells (Figure S3). Indeed, BT549 and SUM159PT cells express higher levels of epithelial-to-mesenchymal transition (EMT) markers, such as N-cadherin, vimentin, Twist, and Zeb1/2, than human ductal MCF10DCIS, which is derived from a nonmalignant immortalized human mammary epithelial cell line and expresses epithelial markers (Figure S4A,B). As shown in Figure S4C, BT549 and SUM159PT cells synthesized and secreted Pn protein in culture medium. Thus, a Pn exon-21 splicing reporter was introduced in these two cell lines (Figure 1A and Figure S5). This reporter allowed us to visualize cells expressing the Pn exon-21 ASV (Figure 1B). Interestingly, the SUM159PT cell line showed a GFP single-positive fraction (exon-21 exclusion) and GFP/mCherry double-positive fraction (Figure 1C), whereas only a double-positive fraction was detected in BT549 cell lines. BT549 and SUM159PT belong to the mesenchymal type (ML) and mesenchymal-stem-like type (MSL) of TNBC. It is reported that the ML and MSL subtypes display a variety of unique gene signatures that are heavily enriched in components and pathways involved in cell motility, ECM receptor interaction, and cell differentiation pathways. However, unique to the MSL are genes representing components and processes linked to growth factor signaling pathways that include inositol phosphate metabolism, EGFR, PDGF, calcium signaling, G-protein coupled receptor, and ERK1/2 signaling, as well as ABC transporter and adipocytokine signaling [13]. This fact suggests that the MSL subtype contains stemlike cells. As periostin is highly expressed in breast cancer stem cells and is known to maintain it [14], SUM159PT TNBC cell lines were intended for further analysis. Following isolation of GFP single-positive and double-positive fractions by FACS, we sought to validate the utility of splicing reporters in tracing cells by quantitative polymerase chain reaction (qPCR). As shown in Figure 1D, the cells from the double-positive fraction expressed significantly higher levels of Pn ASV with exon 21 (Pn 21) than the GFP single-positive fraction. The expression level of Pn ASV without exon 21 (Pn 20/22) was also higher in the double-positive fraction than in the GFP single-positive fraction. Additionally, media from the double-positive fraction contained a larger amount of Pn ASVs (Figure 1E), which could be captured by Pn-21 Ab (Figure 1F). The use of a reporter minigene system has at least two limitations. The first is that splicing happens even in the periostin-negative cells due to the use of CMV promoter. The second is that the insertion of introns that are not complete sequences might not reflect the full splicing mechanism. However, these results indicated that the Pn exon-21 EGFP/mCherry splicing reporter could distinguish between cells with high and low Pn expression.

### 3.2. Characterization of GFP Single- and Double-Positive SUM159PT TNBC Cells

**Figure 2:**
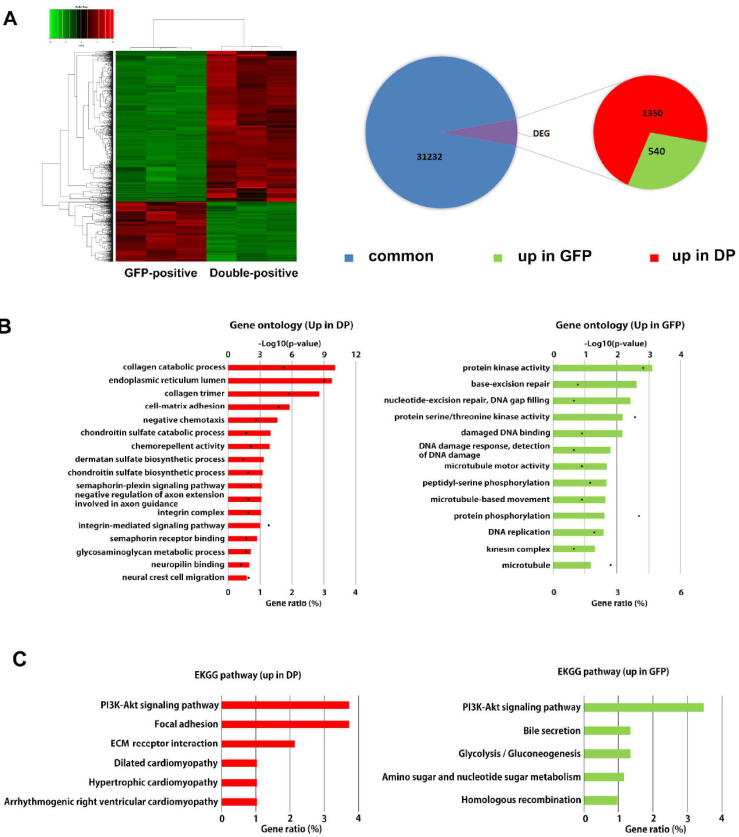

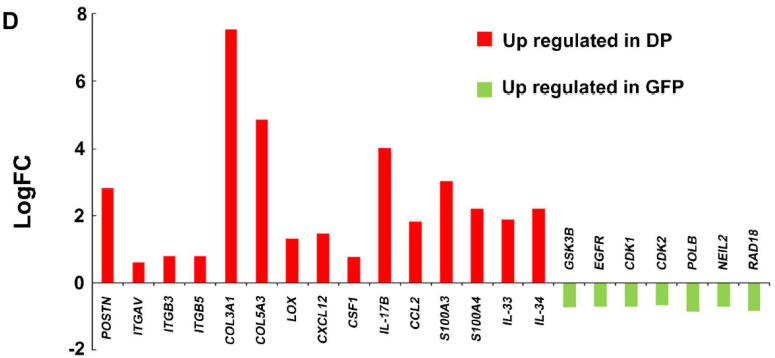
RNA-seq analysis comparing GFP- and double-positive SUM159PT. (**A**) RNA-seq analysis showing a heat map of differentially expressed genes (DEGs) in GFP- and double-positive SUM159PT. The data represent three biological replicates. Among 1890 DEGs, 1350 were upregulated in double-positive cells, while 540 were upregulated in GFP-positive cells. (**B**) Gene ontology enrichment of double-positive cells (DP) and GFP-positive cells (GFP). The lengths of the bars indicate the -log 10-transformed *p*-values. Each dot represents the proportion of genes with the GO term among DEGs. (**C**) KEGG analysis of upregulated genes in the double-positive cells (DP) and the GFP-positive cells (GFP) from the RNA-seq data. Three biological repeats of each experiment were conducted. (**D**) Upregulated genes in double-positive cells (DP) include several genes related to macrophage differentiation and recruitment. Data are the log2 of fold change (LogFC). Relative expression pattern analysis of upregulated genes in DP by qRT-PCR analysis to validate the RNA-seq data is shown in Figure S7.

To characterize the GFP single-positive and double-positive cells, we performed RNA sequencing for gene profiling. The expression profiles of both fractions were concordant (average correlation coefficient 0.98), and 1890 differentially expressed genes (DEGs, FDR < 0.05) out of 33,122 examined genes were detected. Among the DEGs, 1350 genes and 540 genes were upregulated in double-positive and GFP single-positive cells, respectively (Figure 2A and Table S1). DEGs upregulated in double-positive cells were related to collagen catabolic processes, endoplasmic reticulum lumen, collagen trimer, and cell matrix adhesion, whereas DEGs upregulated in GFP single-positive cells were associated with protein kinase activity, base-excision repair, and nucleotide-excision repair (Figure 2B). To further clarify the biological functions of the identified DEGs, pathway-enrichment analysis was performed by analyzing the DEGs using the KEGG pathway database [15]. The enriched pathways in the double-positive cells were associated with the PI3K-Akt signaling pathway, focal adhesion and ECM-receptor interaction, while those of GFP single-positive cells were associated with the PI3K-Akt signaling pathway, bile secretion, and glycolysis-related pathway (Figure 2C). The upregulation of ECM-related genes (e.g., COL3A1, COL5A3, LOX, POSTN) and their receptor genes (e.g., ITGAV, ITGB3, ITGB5) in the double-positive cells indicates that multicomponent networks surround cells that are obligatory for cell survival, growth, and differentiation. These ECM-derived signals are known to be critically involved in the process of EMT during tumorigenesis [16]. Additionally, expression of cytokines such as CXCL12, CSF-1, IL-17B, CCL2, S100A3, S100A4, IL-33 and IL-34, which are known to recruit tumor-associated macrophages (TAMs), was dominant in double-positive cells (Figure 2D and Figure S7). Cell migration ability was comparable between both fractions (Figure S8). These results suggest that SUM159PT TNBC cells contain a subset of cells with high Pn expression accompanied by a mesenchymal-stem-like phenotype with low Pn expression.

### 3.3. Antitumor Activity of Pn-21 Ab in Xenografts of SUM159PT Double-Positive Cells

**Figure 3:**
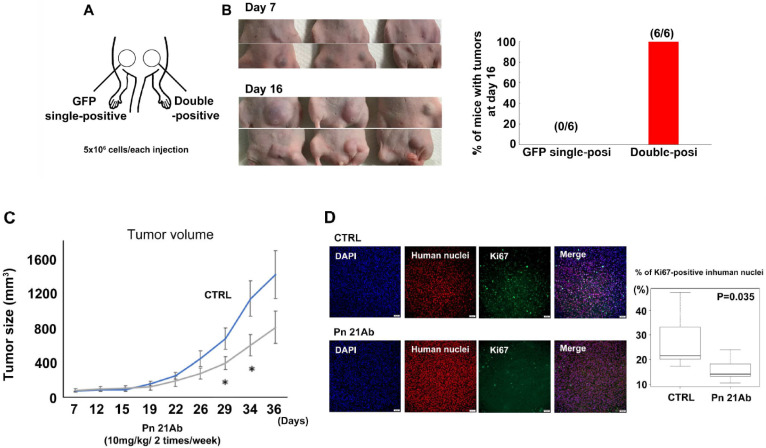
Pn ASV-specific antibody (Pn-21 Ab) inhibited the growth of double-positive SUM 159PT xenografts. (**A**) In total, 5 × 10^6^ cells from SUM159PT GFP- and double-positive cells were separately injected into the abdominal mammary glands of athymic nude mice. (**B**) Images of tumor development at day 7 and 16 post injection. While GFP-positive cells did not form tumors (0/6), double-positive cells engrafted and developed tumors (6/6). (**C**) Athymic nude mice bearing SUM159PT double-positive cell tumors were treated with Pn-21 Ab or vehicle (*n* = 7 for Pn-21 Ab, *n* = 6 for CTRL). The vehicle group received saline, and the other group was treated with Pn-21 Ab (10 mg/kg) 2 times per week throughout the experiments starting at day 0. Tumor volumes were recorded as mean ± SD. * *p* < 0.05. (**D**) Pn-21 Ab reduced proliferation in SUM159PT double-positive cell xenografts. Paraffin-embedded tumor sections from the CTRL and Pn-21 Ab groups were stained with antibodies against Ki67 and human nuclei. Ki67-positive human nuclei were quantified. Representative photomicrographs are presented, and quantitative data of Ki67-positive human nuclei are shown in box plots. The *p* value was calculated using the Mann–Whitney U test. Scale bars represent 50 μm.

To validate the role of Pn in SUM159PT TNBC cell lines, 5 × 10^6^ GFP single-positive cells or double-positive cells were injected subcutaneously into athymic nude mice (Figure 3A). At day 16, all mice (6/6) developed tumors from double-positive cells, while no tumors (0/6) developed from GFP single-positive cells (Figure 3B). Then, we investigated the antitumor activity of Pn-21 Ab in the xenograft model injected with SUM159PT double-positive cells. Pn-21 Ab at a dose of 10 mg/kg was given intravenously by tail vein twice a week throughout the experiment starting at day zero. At day 36, tumor growth was significantly reduced by approximately 40% in Pn-21 Ab-treated mice compared with the control group (*p* < 0.01; Figure 3C). Pn-21 Ab treatment did not cause toxicity in body weight (data not shown). Tumor cells in the cell cycle were assessed by measuring the fraction of Ki-67-positive tumor cells. Administration of Pn-21 Ab significantly decreased the fraction of Ki-67-positive tumor cells compared to the control group (Figure 3D). However, Pn-21 Ab had little effect on the proliferation of SUM159PT double-positive cells in vitro (Figure S9). These data suggest that Pn ASV with exon 21 secreted from cancer cells affect cancer cell growth through an indirect mechanism.

### 3.4. Pn Enhances TAM M2 Polarization

**Figure 4:**
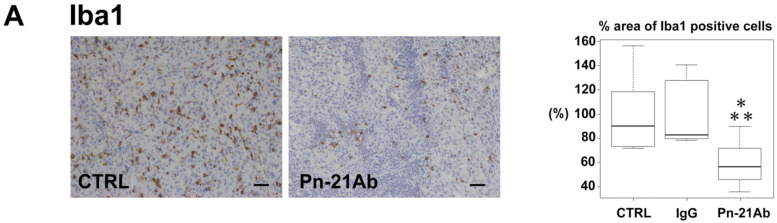

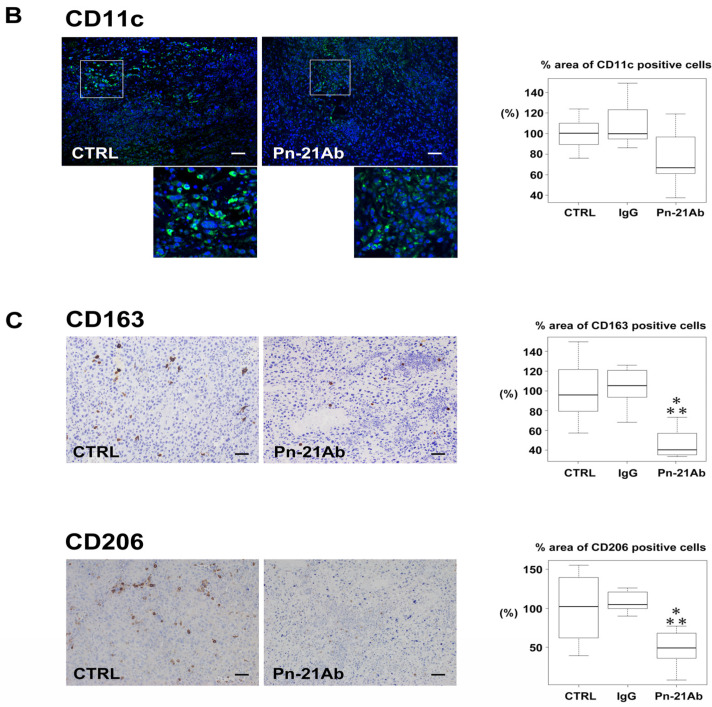
Disrupting Pn ASV with Pn-21 Ab reduces M2 tumor-supportive TAMs in vivo. Immunohistochemical or immunofluorescent staining with the pan macrophage marker (Iba1) (**A**), M1 macrophage marker (CD11c) (**B**), and M2 macrophage markers (CD163 and CD206) (**C**) in SUM159PT double-positive cell xenografts. Tumors were collected at day 36. Scale bars represent 50 μm. Box plot showing a significant reduction in M2 TAM density in Pn-21 Ab treated xenografts (*n* = 6–7). The TAM marker-positive area of tumors was quantitated in at least 4 view fields per tumor by using Image J program. *** *p* < 0.05 vs. CTRL, IgG, respectively. *p* values were calculated by using the one-way ANOVA test and Tukey–Kramer’s post hoc test.

**Figure 5:**
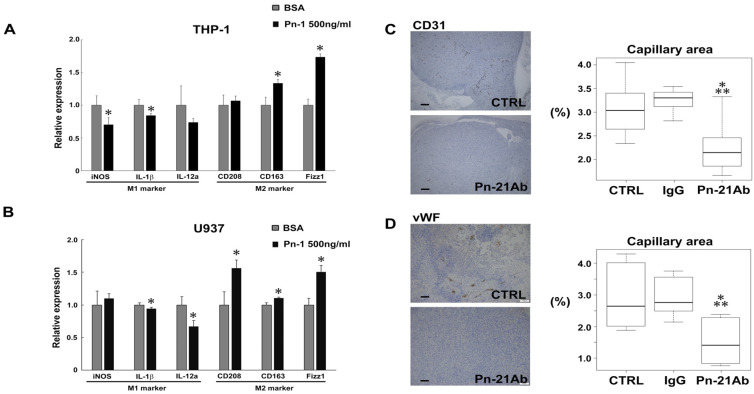
Pn with exon 21 reduces M1 macrophage markers but enhances M2 macrophage markers. U937 M0 macrophages were treated with recombinant full-length Pn protein, Pn-1. (**A**,**B**) RT-qPCR analyses showing upregulation of M2 TAM markers induced by recombinant Pn-1 in THP-1 (**A**) or U937 (**B**) macrophages. PMA-treated macrophages were stimulated with recombinant Pn (500 ng/mL) in RPMI media supplemented with 0.1% BSA for 24 h. RT-qPCR analysis indicated downregulation of M1 markers (iNOS, IL-1β and IL-12a) and up-regulation of M2 markers (CD206, CD163 and Fizz1). Data are shown in mean ± SD, *n* = 4 per group, * *p* < 0.05 vs. BSA. Student’s *t*-test. (**C**,**D**) Immunohistochemical staining of CD31 (**C**) or vWF (**D**), an endothelial cell marker. CD31- or vWF-positive area in tumors was quantitated in at least 4 view fields per tumor by using Image J program. (*n* = 6–7). *, ** *p* < 0.05 vs. CTRL, IgG, respectively. Box plot showing mean % of capillary area in tumors. Scale bars represent 100μm. *p* values were calculated by using the one-way ANOVA test and Tukey–Kramer’s post hoc test.

The RNA-seq data revealed enriched expression of genes related to TAM accumulation in double-positive cells. Recent studies have suggested the existence of at least two subtypes of TAMs in tumors (M1 and M2). The M1 subtype has an inhibitory effect on tumor growth, while the M2 subtype plays a supportive role in tumor progression by secreting cell growth-related cytokines and survival factors [17]. Cell-surface marker Iba1 is commonly used to identify the entire TAM population. CD11c is suggested for M1 TAMs, whereas CD163 and CD206 are suggested for M2 TAMs. Immunohistochemical or immunofluorescent staining of samples from a xenograft model of SUM159PT double-positive cells showed that the pan-TAM marker Iba1-positive TAMs were significantly reduced in tumors treated with Pn-21 Ab (Figure 4A). The M1 macrophage marker CD11c-positive TAMs were slightly but not significantly decreased in the Pn-21 Ab treated group (Figure 4B). Interestingly, M2 macrophage marker CD163- or CD206-positive TAMs were significantly decreased in Pn-21 Ab-treated tumors (Figure 4C). These data suggest that Pn ASV with exon 21 secreted in the stroma stimulates TAMs, enhancing M2 polarization and possibly enhancing tumor growth. Indeed, it has been demonstrated that a higher number of M2 TAMs in breast cancer correlates with higher expression of the tumor proliferation marker Ki-67 [18]. Moreover, compelling evidence has shown that TAMs abundant in the microenvironment of breast cancers are generally M2-polarized. To date, M2-polarized macrophages have been found to be associated with poor prognoses in various cancers [19]. To confirm the effect of Pn with exon 21 on TAM polarization, PMA-primed M0 THP-1, U937 macrophages and GM-CSF-treated human CD14+ PBMCs were treated with recombinant human Pn with exon 21 (Pn-1) or Pn lacking exon 17 and 21 (Pn-4). Pn-1 treatment significantly enhanced the expression of M2 TAM markers, such as CD206, CD163 and Fizz1, but reduced the M1 TAM markers iNOS, IL-1β and IL-12a (Figure 5A,B and Figure S10). However, Pn-4, which lacks periostin exon 21, did not enhance M2 TAM polarization (Figures S10 and S11). It is important to mention that this Pn-21 Ab has no effect on macrophage polarization (Figure S12A), and isotype control (IgG2b) has no effect on tumor growth in vivo (Figure S12B), indicating that the change of M1 and M2 TAM markers were dependent on the periostin splicing variant. Finally, we asked whether Pn-21 Ab could reduce tumor angiogenesis since we previously demonstrated that Pn ASVs with exon 21 enhances angiogenesis in an in vitro tube formation assay [6], and that M2-polarized TAMs secrete angiogenic growth factors. Moreover, SUM159PT double-positive cells showed higher expression of angiogenic factors, such as Angiopoietin 1, HGF, TGFβ2, PGF and MMP2. As expected, CD31 and von Willebrand factor (vWF) staining revealed that the capillary density in tumors was reduced significantly in the Pn-21 Ab-treated group compared to the saline control group and isotype IgG group (Figure 5C,D). The finding that treatment with Pn-21 Ab significantly reduced M2 TAM polarization, angiogenesis and subsequent TNBC cell growth in mice was in accordance with the fact that the Pn gene is a major actor in maintaining the cancer microenvironment and facilitates tumor growth by modulating the immune system and angiogenesis.

## 4. Discussion

TNBC is a subtype with distinct heterogeneity, higher invasiveness, and poorer prognosis due to lack of target molecules [20]. Thus, discovery of new therapeutic targets and effective therapeutic agents is urgently needed. In the present study, we found that the SUM159PT TNBC cell line contains two subpopulations: cells secreting large amounts of Pn (Pn-positive cells) and those secreting negligible amounts of Pn (Pn-negative cells). By RNA sequencing, we revealed that Pn-positive cells are enriched in genes related to focal adhesions (e.g., TLN2, VCL, MYLK2, MYLK4, FLNB, LAMC1, LAMC2, LAMC3), ECM-receptor interactions (e.g., COL1A1, COL3A1, COL5A2, COL6A1, ITGA1, ITGA10, ITGA11, ITGB3, ITGB5, ITGB8, LAMA1, LAMB1) and the PI3K-Akt signaling pathway (e.g., LPAR4, TLR4, LPAR1, ANGPT1, PDGFD, VEGFC, PDGFRA, PDGFRB CSF1, KITLG) compared to Pn-negative cells. Focal adhesions are large structures through which integrins and scaffold proteins link the actin cytoskeleton to the ECM in many cell types. Focal adhesions and ECM-receptor interactions are essential parts of the tumor microenvironment [21,22]; they provide structural support, supply growth and survival signals, and control invasion by tumor cells, as well as by immune cells, fibroblasts and microvessels, thereby promoting the progression of the tumor [23,24]. Indeed, in our study, only Pn-positive SUM159PT cells were able to engraft and form tumors, suggesting the growth benefit characteristics of Pn-positive SUM159PT cells in a mouse xenograft model. Lehmann et al. described six distinct subtypes of TNBC according to gene expression profiling: basal-like (BL1 and BL2), immunomodulatory (IM), mesenchymal (M), mesenchymal stem–like (MSL), and luminal androgen receptor (LAR) subtypes [13]. The MSL subtype to which the SUM159PT cell line belongs is enriched with components and pathways involved in ECM receptor interactions, cell differentiation pathways (Wnt pathway, anaplastic lymphoma kinase pathway, and TGF-β signaling), growth-factor signaling pathways, ABC transporter and adipocytokine signaling and angiogenesis, as well as immune signaling [13]. MSL is also enriched in the expression of genes associated with stem cells (ABCA8, ABCB1, ALDHA1, PER1, BMP2, and THY1), numerous HOX genes (HOXA5, HOXA10, MEIS1, MEIS2, MEOX1, MEOX2, and MSX1), and mesenchymal stem cell-specific markers (BMP2, ENG, ITGAV, KDR, NGFR, NT5E, PDGFRB, THY1, and VCAM1) [13]. Interestingly, the levels of these MSL-related genes such as ABCA6, ABCA8, ABCA9, were mostly higher in Pn-positive cells than in Pn-negative cells (Table S1). These data indicate that Pn-positive cells underlie the MSL gene signature of SUM159PT cells and this might be the reason that SUM159PT cells contains two populations: Pn-positive and Pn-negative.

Pn has been demonstrated to regulate multiple biological aspects of tumor cells, including proliferation, invasion, survival, angiogenesis, metastasis and resistance to chemotherapy [25,26,27,28]. Moreover, Pn plays a pivotal role in remodeling various tumor microenvironments, such as the cancer stem-cell niche, perivascular niche, premetastatic niche, and immunosuppressive microenvironment [29]. In the present study, we demonstrated that Pn-21 Ab reduced M2 TAM polarization and angiogenesis in tumors. Additionally, in vitro experiments revealed that Pn with exon 21 could reduce the expression of M1 TAM markers (iNOS and IL-12a) but enhance the expression of M2 markers (CD163, CD206, Fizz 1), whereas physiological Pn ASVs did not change TAM polarization. TAMs are the most abundant inflammatory cells in breast cancers and the association between the high influx of TAMs in tumors and poor prognosis have been demonstrated clinically. In tumors, TAMs either enhance (M1 TAMs) or antagonize (M2 TAMs) the antitumor efficacy of cytotoxic agents, antibody-targeting cancer cells, and therapeutic agents by producing high levels of anti-inflammatory and angiogenic factors [30]. Therefore, strategies targeting M2 TAMs are greatly promising therapies that involves altering the immunosuppressive tumor microenvironment for enhanced cancer therapy. RNA-seq data also demonstrated that several genes related to adoptive immune systems which have pro-tumor action, such as TGFβ2, TGFBI, TGFBR1, TGFBR3, PLAU, IL-1β, and IL-33 are higher in Pn-positive cells than Pn-negative cells. Additionally, Pn is known to promote myeloid-derived suppressor cells (MDSC)-mediated pulmonary premetastatic niche formation during breast tumor metastasis through S100A8/9 and LOX [31]. Interestingly, these genes are also upregulated in Pn-positive fraction, suggesting Pn-21 Ab might reduce tumor growth through suppression of MDSC. It is also important to mention that paclitaxel, which is often used in the treatment of recurrent TNBC, had no effect on the SUM159PT Pn-positive cell xenograft (Figure S13A). Moreover, paclitaxel significantly increased Pn secretion in SUM159PT double-positive cells in vitro (Figure S13B), suggesting that TNBC cells stimulated by conventional chemotherapy might create an environment that supports TNBC cells through Pn enhancing chemoresistance.

Pn is known to be the factor that recruits and polarizes TAMs, causing chemoresistance in pancreatic cancer and glioblastoma [28,32]. Although the signaling cascade regulating TAM polarization by Pn ASV with exon 21 is largely unknown, and even the mechanism that causes Pn ASV with exon 21 in TNBC is unidentified, the targeting of pathological ASVs with Pn-21 Ab might offer a safe and effective strategy for the treatment of TNBC.

## 5. Conclusions

TNBC-derived Pn ASV educates TAMs and regulates angiogenesis, which in turn establishes a microenvironmental niche supportive of TNBC.

## Figures and Tables

**Table 1:** Human primer list.

Name	Forward (5′ to 3′)	Reverse (5′ to 3′)
periostin exon 21	GGTCACCAAGGTCACCAAATTC	CCTGAAGTCAACTTGGCTCTCAC
periostin exon20/22	GTTACAAGAAGACACACCCGTG	CCTGAAGTCAACTTGGCTCTCAC
GAPDH	GGATTTGGTCGTATTGGG	GGAAGATGGTGATGGGATT
ITGAV	AGGAGAAGGTGCCTACGAAGCT	GCACAGGAAAGTCTTGCTAAGGC
ITGB3	CATGGATTCCAGCAATGTCCTCC	TTGAGGCAGGTGGCATTGAAGG
ITGB5	GCCTTTCTGTGAGTGCGACAAC	CCGATGTAACCTGCTGGCACT
COL3A1	TGGTCTGCAAGGAATGCCTGGA	TCTTTCCCTGGGACACCATCAG
COL5A3	GAGAGGAGAACTGGGCTTCCAA	TAGAGGTCCCACTTCTCCTGTC
LOX	ACAATGTTGTGCGCTGTGAC	CCACTTCAGAACACCAGGCA
CXCL12	CTCAACACTCCAAACTGTGCCC	CTCCAGGTACTCCTGAATCCAC
CSF1	TGAGACACCTCTCCAGTTGCTG	GCAATCAGGCTTGGTCACCACA
IL-17A	GCTGTGGATGTCCAACAAGAGG	TCCTGCATGGTGAAGGGGTTCA
CCL2	CCCCAGTCACCTGCTGTTAT	TGGAATCCTGAACCCACTTC
S100A3	CAAATACAAGCTCTGCCAGGCG	TCGCAGTCCTTGTTGGTGTCCA
S100A4	CAGAACTAAAGGAGCTGCTGACC	CTTGGAAGTCCACCTCGTTGTC
IL-33	GCCTGTCAACAGCAGTCTACTG	TGTGCTTAGAGAAGCAAGATACTC
IL-34	CCAAGGTGGAATCCGTGTTGTC	CACCTCACAGTCCTGCCAGTTT
E-cadherin	CTGAGAACGAGGCTAACG	TTCACATCCAGCACATCC
N-cadherin	GCTGATAGCCCGGTTTCACT	CCCAGGCTTTGATCCCTCTG
Vimentin	GGACCAGCTAACCAACGACA	AAGGTCAAGACGTGCCAGAG
Twist	TACGCCTTCTCGGTCTGGA	ACTGTCCATTTTCTCCTTCTCTGG
Snail 1	GAGCTGACCTCCCTGTCAGA	GGCCTCCAAGGAAGAGACTG
Snail 2	GGCTGGCCAAACATAAGCAG	TTGCCGCAGATCTTGCAAAC
Zeb 1	GTGGCGGTAGATGGTAAT	CTGTTTGTAGCGACTGGA
Zeb 2	ACCAGCCCTTTAGGAGTT	AGACCGACAGGCGGAATA
iNOS	GCTCTACACCTCCAATGTGACC	CTGCCGAGATTTGAGCCTCATG
IL-1b	CCACAGACCTTCCAGGAGAATG	GTGCAGTTCAGTGATCGTACAGG
IL-12a	TGCCTTCACCACTCCCAAAACC	CAATCTCTTCAGAAGTGCAAGGG
CD206	CTCTGTTCAGCTATTGGACGC	CGGAATTTCTGGGATTCAGCTTC
CD163	CCAGAAGGAACTTGTAGCCACAG	CAGGCACCAAGCGTTTTGAGCT
Fizz1	GCAAGAAGCTCTCGTGTGCTA	AACATCCCACGAACCACAGCCA

## Data Availability

The data presented in this study are available on request from the corresponding author.

## Supplementary Materials

The following are available online at https://www.mdpi.com/article/10.3390/cancers13205072/s1, Figure S1: Periostin alternative splicing variants, Figure S2: Periostin expression in several breast cancer cell lines, Figure S3: E-cadherin and N-cadherin expression in several breast cancer cell lines, Figure S4: Periostin and EMT marker gene expression in triple-negative breast cancer cell lines, Figure S5: Periostin genomic sequence used for splicing reporter, Figure S6: BT549 with periostin splicing reporter, Figure S7: Representative genes highly expressed in double-positive SUM159PT cells, Figure S8: Cell migration. Cell migration was measured by Oris™ Cell Migration Assays. *n* = 8. NS means not significant, Figure S9: Pn-21 Ab has no effect on SUM159PT double-positive cell proliferation, Figure S10: Pn-4 dose not enhance M1 or M2 TAM polarization, Figure S11: Pn-4 dose not enhance M1 or M2 TAM polarization. PMA-primed M0 macrophages (THP-1 and U937) were treated with Pn-4 (500 ng/mL) for 24 h. M1 and M2 TAM markers were analyzed by quantitative RT-PCR. *n* = 3, * *p* < 0.05 vs. BSA, Figure S12: Effect of Pn-21 Ab on TAM polarization in vitro and control IgG on tumor growth in vivo. GM-CSF-primed human CD14+ PBMNCs were treated with Pn-21Ab (1 or 10 ug/mL). The expression of M1 and M2 macrophage markers were measured by quantitative RT-PCR. *n* = 4. Pn-21 Ab has no effect on macrophage polarization (A). Additionally, isotype control IgG (10 mg/kg/time, 2 times per week) has no effect on tumor growth in SUM159PT xenograft model (B), Figure S13: Effect of Paclitaxel (PTX) on SUM159PT double positive cells in vivo and in vitro. (A) PTX 10mg/kg/week administration has no effect on SUM159PT double positive cell growth in vivo. (B) PTX (10 nM) treatment significantly increases secretion of periostin in vitro, Table S1: RNA-seq results from double-positive and GFP-positive SUM159PT cells.
