# Supplementary material for: Periostin Exon-21 Antibody Neutralization of Triple-Negative Breast Cancer Cell-Derived Periostin Regulates Tumor-Associated Macrophage Polarization and Angiogenesis
Source: Cancers (Basel). 2021 Oct 11;13(20):5072. doi: 10.3390/cancers13205072 (PMC8533925; doi:10.3390/cancers13205072)
Supplement: Supplementary file 1 [file cancers-13-05072-s001.zip › cancers-1362857-supplementary.pdf]

Supplementary materials

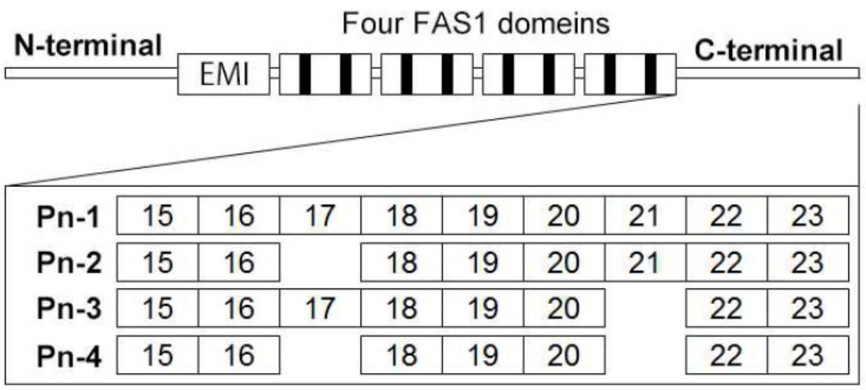

Figure S1. Periostin alternative splicing variants. Periostin has four major alternative splicing variants in its c-terminal region.

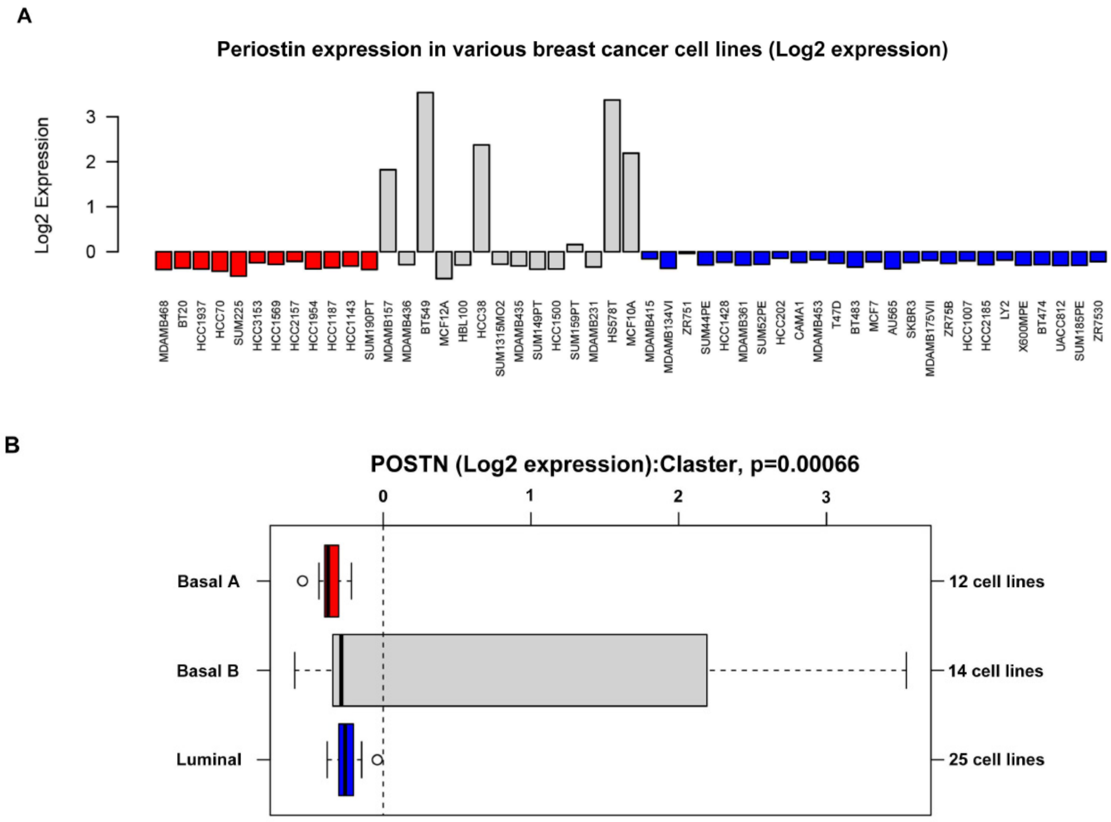

Figure S2. Periostin expression in several breast cancer cell lines. **(A)** Periostin expression was confirmed in several human breast cancer cell lines based on dta available in the GOBO database. **(B)** Periostin expression in the claster of Basal A, Basal B, and Lumnal type breast cancer cell lines was shown. Periostin expressing cells are exclusively limited to the Basal B TNBC cell lines characterized by EMT phenotype.

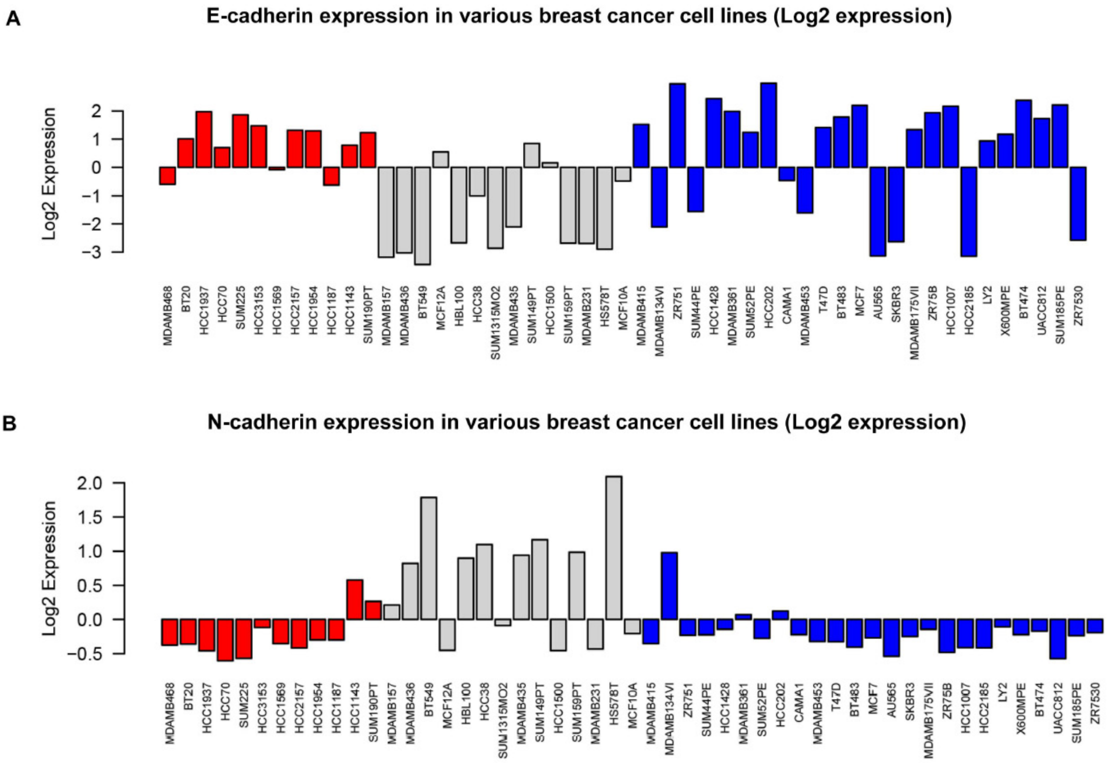

Figure S3. E-cadherin and N-cadherin expression in several breast cancer cell line**.** E-cadherin (A) and N-cadherin (B) expression was confirmed in several human breast cancer cell lines based on data available in the GOBO database. Cells expressing periostin are characterized by EMT phenotype.

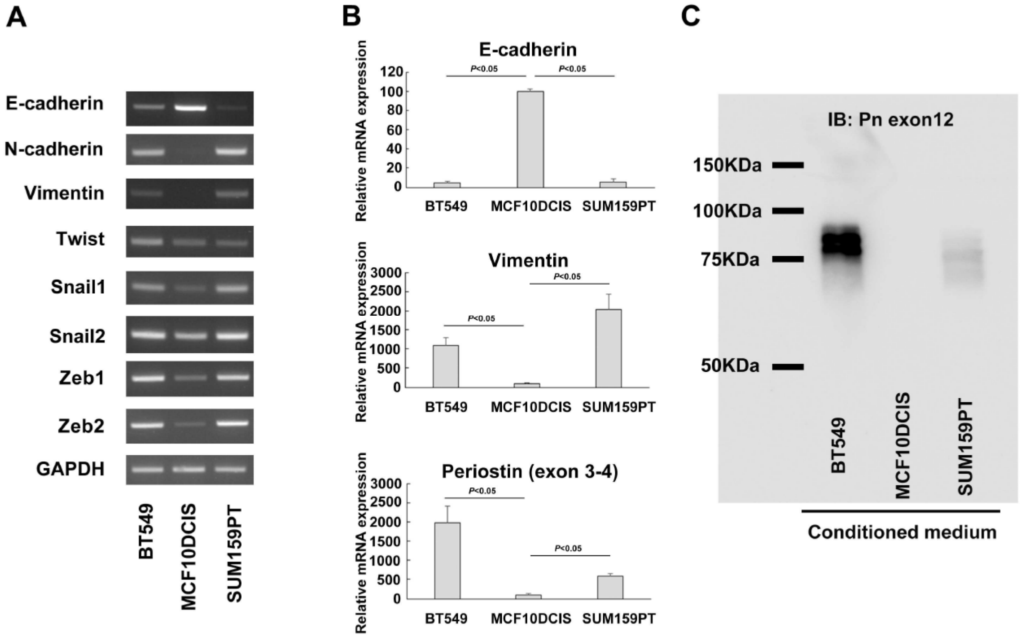

Figure S4. Periostin and EMT marker gene expression in triple negative breast cancer cell lines. **(A)** EMT marker gene was measured by RT-PCR performed on RNA extracted from BT549, MCF10 DCIS, and SUM 159PT cell. **(B)** E-cadherin, vimentin, and periostin mRNA was measured by real time PCR. n=3. **(C)** Western blotting for periostin (exon 12) from conditioned medium was performed on BT549, MCF10 DCIS, and SUM 159PT cell. 5ug protein from BT549, 50ug protein of conditioned media from MCF10DCIS and SUM159PT was loaded.

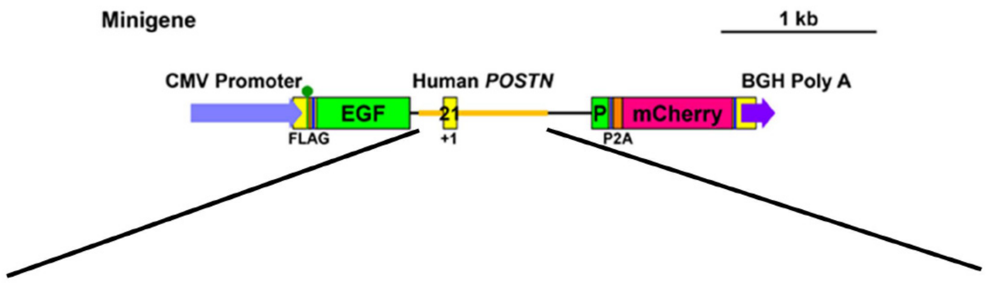

GAATATTAGTATGTCAGAAAACAGCACTAAAATTTGTGAAAGAATCGAGTGTTGGGCTGGAATTCAT
TATTGGGCAGATGCCATTATTTATGACATAAAGTCTGCTGTCTTTATTTTGTTATATAAACCAATTTCT
GCTATAAACATGTATCCTCTCAGAGGTCACCAAGGTCACCAAAATTCATTGAAGGTGGTGATGGTCA
TTTATTTGAAGATGAAGAAATTAAAAGACTGCTTCAGGGAGGTTAGTAAAAGGACAACAACTAACCC
CCTTAACAGGTTCTAGTGACTGAGTTCAACTTTTTAAAGACTGAGCAAGCAGATTTTTTCTTTTAATTG
GGTTAAAAAAAAAAAAAGACAACATCCACTGTTGTCTTAATAGATGGATTGGTTCAATGGTAGTTAATA
GTTATGAAGAAATGATTACTAGTTTAAACAGTCTTTAAAAAGTTCTATGGTATAAAGTTTGGGGAGCA
ATGAATATTTTTTTTGCTTTGATTCAGATAATTTTAAGGGCCTTTTAAGTATGCAAAAGGGAAATAATT
GTTAAATTGCTAGCTGTTAAACAAGCCCAAATTTGATATACTTTTTATATTTAAAAAATTATATTCACTG
CCCCCATAAGAGCAATCAAGGCATGTCTTTAAATTCTATACATAGATATAGCCAAAAATAGTGCATTT
AGTAACATTCTTTTCCAAAACTATATTCTTGGGAATGAATATCTGTTTCTTCTAACAGTTTGAGTGATA
ATCTATACCTGTAGATATAAGTTATTTTGCATATAAAATTAATCTTAATCTTTTATGGAATGTTCTCTGT
CTGTGGCA

Figure S5. Periostin genomic sequence used for splicing reporter. Human periostin exon 21(red letter) and adjacent intron (black letter) used for splicing reporter is shown. The sequences of exon 21 was modified by insertion of a nucleotides (blue letter).

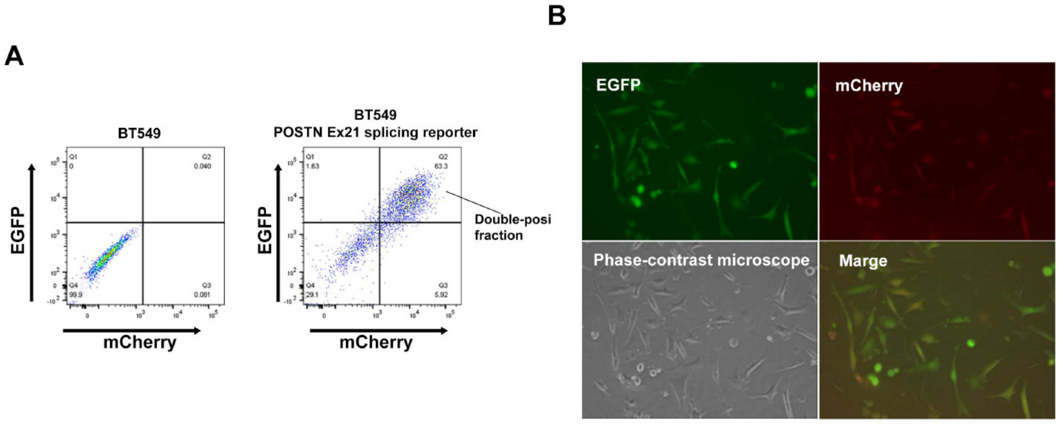

Figure S6. BT549 with periostin splicing reporter. (A) FACS analysis showed that BT549 with periostin splicing reporter demonstrated only GFP- and mcherry double-positive fraction. (B) Fluorescent microscopic and phase-contract microscope image.

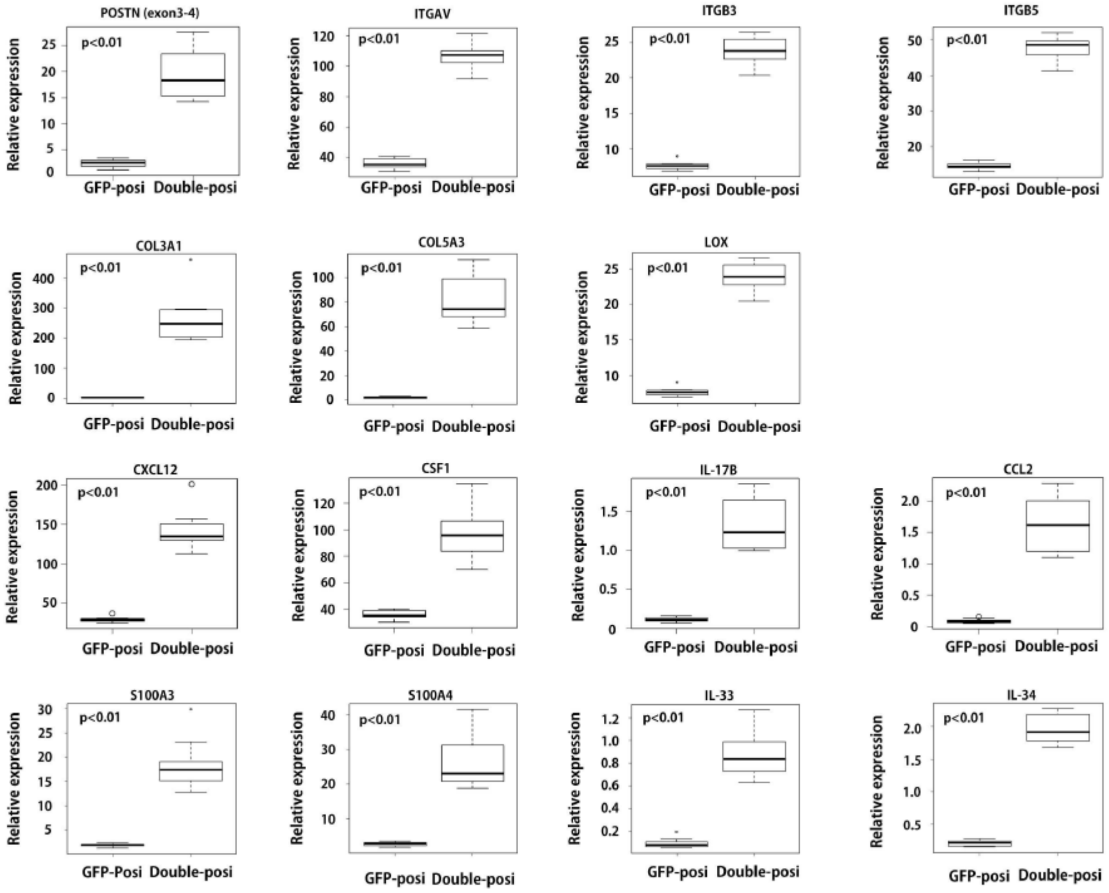

Figure S7. Representative genes highly expressed in double-positive SUM159PT cells**.** Representative genes up regulated in double positive SUM159PT cells were confirmed by RT-PCR. n=10.

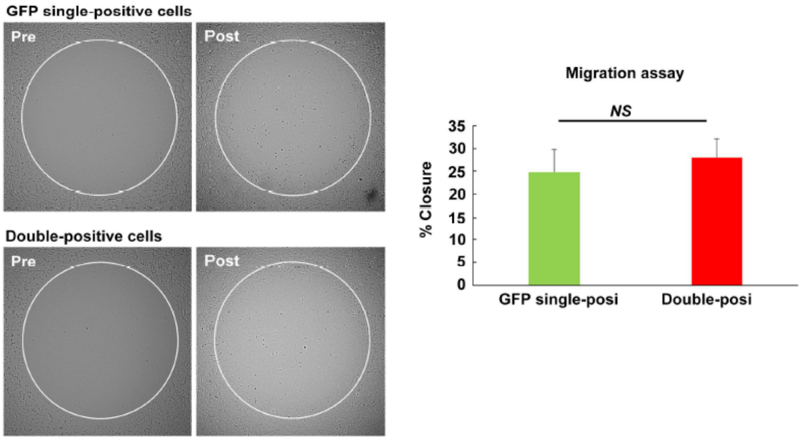

Figure S8. Cell migration. Cell migration was measured by Oris™ Cell Migration Assays. n=8. NS means not significant.

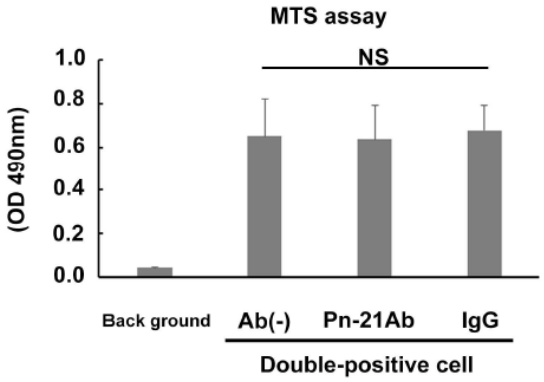

Figure S9. Pn-21Ab has no effect on SUM159PT double-positive cell proliferation. Cell proliferation was measured by MTS assay. SUM159PT double-positive cells were incubated with 20µg/mL Pn-21Ab or control IgG for 24 hr. n=8 NS means not significant.

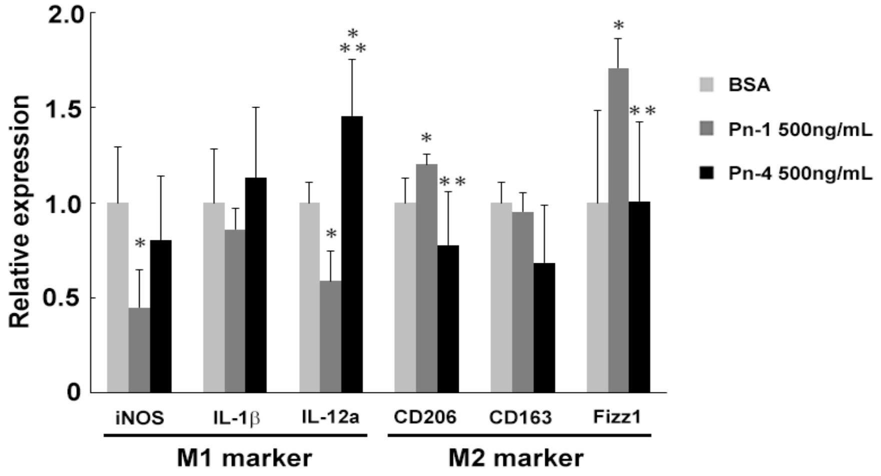

Figure S10. CD14+PBMC primed with 10 ng/mL GM-CSF were treated with Pn-1 (500ng/mL) or Pn-4 (500ng/mL) for 24hrs. M1 and M2 TAM markers were analyzed by quantitative RT-PCR. n=4, ＊, ＊＊P<0.05 vs BSA, Pn-1, respectively.

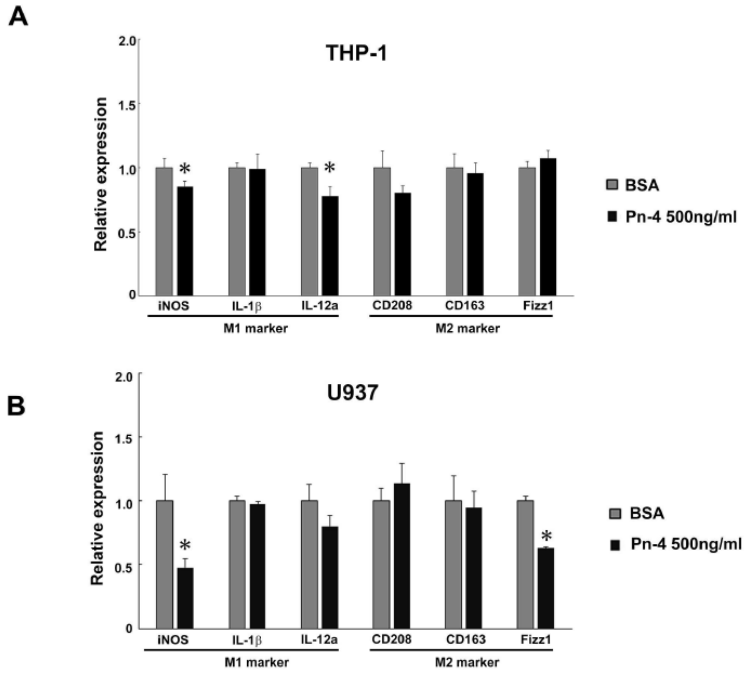

Figure S11. Pn-4 dose not enhance M1 or M2 TAM polarization. PMA-primed M0 macrophages (THP-1 and U937) were treated with Pn-4 (500ng/mL) for 24 hrs. M1 and M2 TAM markers were analyzed by quantitative RT-PCR. n=3, ＊P<0.05 vs BSA.

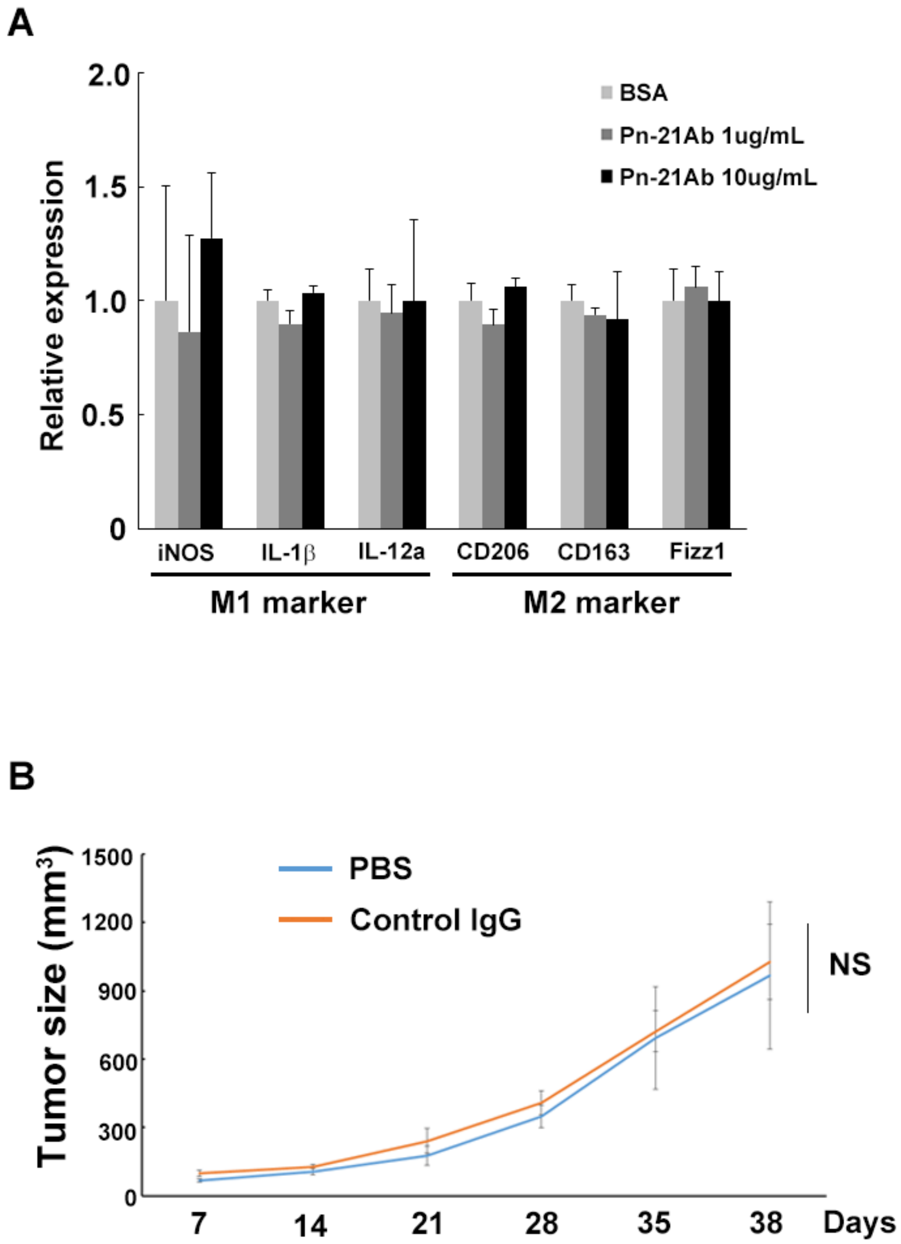

Figure S12. Effect of Pn-21 Ab on TAM polarization in vitro and control IgG on tumor growth in vivo. GM-CSF-primed human CD14+ PBMNCs were treated with Pn-21Ab (1 or 10ug/ml). The expression of M1 and M2 macrophage markers were measured by quantitative RT-PCR. n=4. Pn-21 Ab has no effect on macrophage polarization (A). Additionally, isotype control IgG (10mg/kg/time, 2 times per week) has no effect on tumor growth in SUM159PT xenograft model (B).

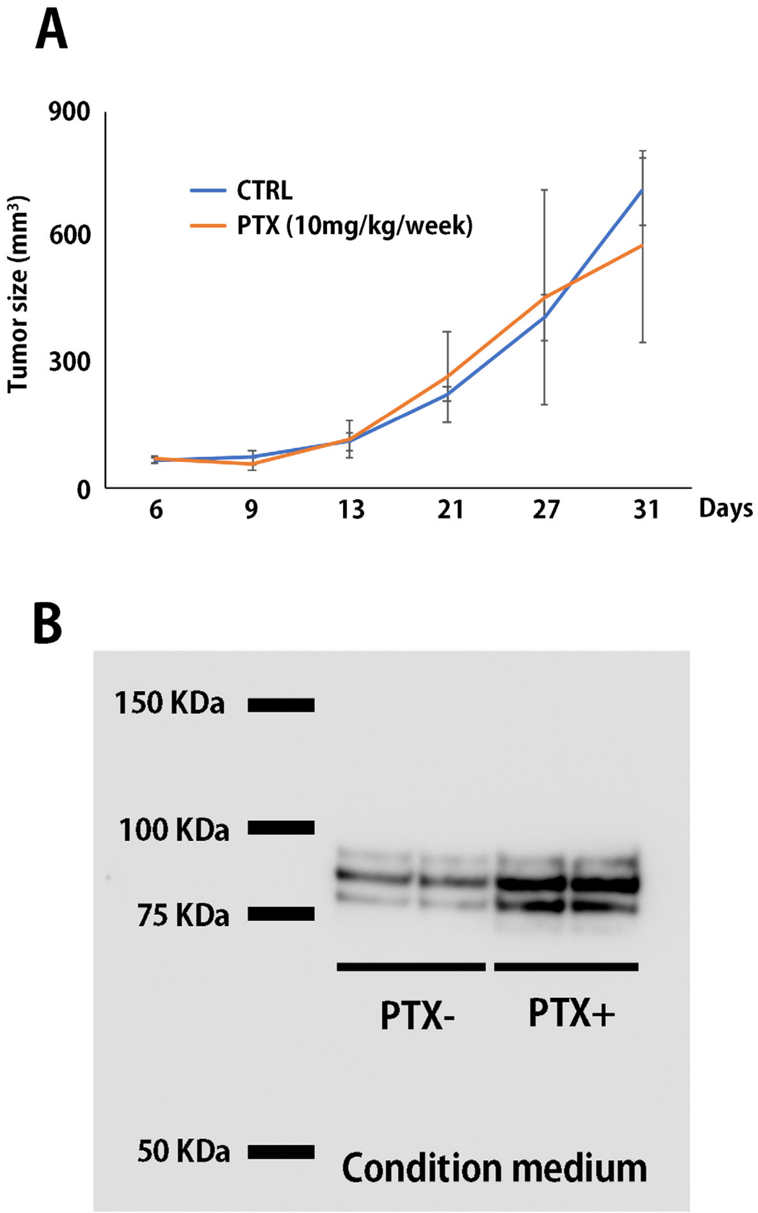

Figure S13. Effect of Paclitaxel (PTX) on SUM159PT double positive cells in vivo and in vitro. (A) PTX 10mg/kg/week administration has no effect on SUM159PT double positive cell growth in vivo. (B) PTX (10nM) treatment significantly increases secretion of periostin in vitro.
